# The Experience of Depression in Older Adults With Cancer: A Qualitative Analysis

**DOI:** 10.1093/geroni/igaf122.761

**Published:** 2025-12-31

**Authors:** Madeleine Hardt, Jaime Gilliland, Lisbeth Rubi, Rinchen Dolma, Jo Anne Sirey, Kelly McConnell

**Affiliations:** Stony Brook Medicine, New York, United States; Memorial Sloan Kettering Cancer Center, New York, New York, United States; Memorial Sloan Kettering Cancer Center, New York, New York, United States; Memorial Sloan Kettering Cancer Center, New York, New York, United States; Weill Cornell Medical College, New York City, New York, United States; Memorial Sloan Kettering Cancer Center, New York, New York, United States

## Abstract

Older adults (≥65 years) will make up 73% of U.S. cancer survivors by 2040. Over 25% of older adults with cancer (OACs) report significant depressive symptoms, linked to worse outcomes. Yet, little is known about OACs’ depression experience. This study explored OACs’ perspectives via focus groups (n = 13 patients receiving or within 6 months of receiving active treatment). Five key themes emerged: (1) OACs conceptualized their depressive symptoms within the context of their cancer experience (e.g., fear of treatment); (2) Depressive symptoms impact daily life (e.g., reduced activity engagement); (3) OACs perceive their depressive symptoms as distinct from clinical depression and “not bad enough” to warrant treatment; (4) OACs prefer self-reliance or support networks over mental health services; (5) Barriers (e.g., appointment burden) limit access to mental health services. Findings highlight OACs’ unique views on depression and suggest that their preference for personal coping strategies may impact mental health service engagement.

